# Two new species of Trichoceridae from the Middle Jurassic Jiulongshan Formation of Inner Mongolia, China

**DOI:** 10.3897/zookeys.411.6858

**Published:** 2014-05-28

**Authors:** Fei Dong, Chungkun Shih, Dong Ren

**Affiliations:** 1 College of Life Sciences,; 2 Capital Normal University, 105 Xisanhuanbeilu, Haidian District, Beijing 100048, China

**Keywords:** Diptera, *Eotrichocera*, *Archaeotrichocera*, Fossil, Daohugou

## Abstract

Two new species, *Eotrichocera* (*Archaeotrichocera*) *longensis* **sp. n.** and *Eotrichocera* (*Archaeotrichocera*) *amabilis* **sp. n.** of Trichoceridae are described based on a combination of the following characters: Sc ending proximad of the forking of R_2_, shape of d cell and A_2_ rather short and bending sharply toward posterior margin. These fossil specimens were collected from the Middle Jurassic Jiulongshan Formation of Daohugou in Inner Mongolia, China.

## Introduction

Trichoceridae is a family of medium-sized dipterans, commonly called winter crane flies, Yang and Yang (1995) found that just a few adults can live in cold environment, even in winter, and indicated that the name of winter crane flies might not be proper. However, the adults (include the largest species of *Trichocera*) not only live in cold environment, but also mate and lay eggs under the snow cover in winter (Hågvar and Krzemińska 2007). Hence, the common name of winter crane flies is proper. The adults live in damp places close to lakes, rivers, or streams and most of them feed on plant fluids (Yang 2009); while the larvae live in moist or wet or terrestrial biotopes and feed on plant debris, decaying leaves in forests, mushrooms and animal droppings (Dahl and Alexander 1976) or take cankered plants or animal bodies as food (Yang 2009).

There are 77 species of fossil and amber trichocerids, which have been assigned into three subfamilies: Trichocerinae, Paracladurinae and Kovalevinae; and twelve genera: *Cladoneura* Scudder, 1894; *Eotrichocera* Kalugina, 1985; *Rasnitsynina* Krzemińska, Krzemiński & Dahl, 2009; *Mailotrichocera* Kalugina, 1985; *Paleotrichocera* Kalugina, 1986; *Karatina* Krzemińska, Krzemiński, Dahl & Lukashevich, 2009; *Trichocera* Meigen, 1803; *Tanyochoreta* Zhang, 2006; *Zherikhinina* Krzemińska, Krzemiński & Dahl, 2009; *Undaya* Krzemińska, Krzemiński & Dahl, 2009; *Kovaleva* Krzemińska, Krzemiński & Dahl, 2009; *Paracladura* Brunetti, 1911 (Krzemińska et al. 2009a). The oldest species of trichocerids, *Mailotrichocera variabilis*, *Mailotrichocera mikereichi* and *Mailotrichocera zessini* have been described from Lower Jurassic of Germany (Krzemińska et al. 2009a).

Among them, there are eight species in three genera described from the Daohugou locality of China: *Eotrichocera (Archaeotrichocera) ephemera* Zhang, 2006; *Tanyochoreta integera* Zhang, 2006; *Tanyochoreta chifengica* Zhang, 2006; *Tanyochoreta (Sinotrichocera) parva* Zhang, 2006; *Eotrichocera (Archaeotrichocera) conica* Krzemińska, Krzemiński & Ren, 2009; *Eotrichocera (Archaeotrichocera) rara* Krzemińska, Krzemiński & Ren, 2009; *Eotrichocera (Archaeotrichocera) spatiosa* Liu, Shih & Ren, 2012 and *Zherikhinina reni* Krzemińska, Krzemiński & Dahl, 2009.

Furthermore, *Sinotrichocera* Zhang, 2006 has been changed as a subgenus belonging to *Tanyochoreta*; *Oligotrichocera* Dahl, 1971 as a subgenus belonging to *Trichocera* Podenas, 2001; *Trichonomites* Kalugina, 1986 and *Paleotrichocera* Kalugina, 1986 are synonymized (Krzemińska et al. 2009a). All genera and species of Trichoceridae Kertész, 1902, after revisions and transfers, are summarized in Table 1, which is updated and expanded from the Tables 1 and 4 in Krzemińska et al. 2009a.

**Table 1.** Fossil species of Trichoceridae Kertész, 1902.

Genus	Species	Author(s)	Date	Age	Locality
*Cladoneura*	*Cladoneura willistoni*	Scudder	1894	Lower Oligocene	Florissant, USA
*Eotrichocera (Archaeotrichocera)*	*Eotrichocera (Archaeotrichocera) ephemera*	Zhang	2006	Middle Jurassic	Daohugou, China
	*Eotrichocera (Archaeotrichocera) conica*	Krzemińska, Krzemiński & Ren	2009a	Middle Jurassic	Daohugou, China
	*Eotrichocera (Archaeotrichocera) rara*	Krzemińska, Krzemiński & Ren	2009a	Lower Cretaceous	Kempendyay, Russia
	*Eotrichocera (Archaeotrichocera) spatiosa*	Liu, Shih & Ren	2012a	Middle Jurassic	Daohugou, China
*Eotrichocera (Eotrichocera)*	*Eotrichocera (Eotrichocera) christinae*	Kalugina	1985	Lower Jurassic or earlier Middle Jurassic	Novospasskoe, Russia
*Karatina*	*Karatina longipes*	Rohdendorf	1964	Upper Jurassic	Karatau, Kazakhstan
	*Karatina explorans*	Krzemińska, Krzemiński, Dahl & Lukashevich	2009a	Lower Cretaceous	Baissa, Russia
	*Karatina pellita*	Krzemińska, Krzemiński, Dahl & Lukashevich	2009a	Upper Jurassic	Karatau, Kazakhstan
*Kovaleva (Kovaleva)*	*Kovaleva (Kovaleva) fragmentosa*	Krzemińska, Krzemiński & Dahl	2009a	Jurassic/Cretaceous boundary	Unda and Daya, E. Transbaikalia
	*Kovaleva (Kovaleva) hirsuta*	Krzemińska, Krzemiński & Dahl	2009a	Jurassic/Cretaceous boundary	Daya, E. Transbaikalia
	*Kovaleva (Kovaleva) obscura*	Krzemińska, Krzemiński & Dahl	2009a	Jurassic/Cretaceous boundary	Daya, Unda and Shevia, E. Transbaikalia
	*Kovaleva (Kovaleva) sheviae*	Krzemińska, Krzemiński & Dahl	2009a	Jurassic/Cretaceous boundary	Shevia, E. Transbaikalia
	*Kovaleva (Kovaleva) volodii*	Krzemińska, Krzemiński & Dahl	2009a	Jurassic/Cretaceous boundary	Daya, E. Transbaikalia
*Kovaleva (Vladimirevna)*	*Kovaleva (Vladimirevna) mirabilis*	Krzemińska, Krzemiński & Dahl	2009a	Jurassic/Cretaceous boundary	Daya, E. Transbaikalia
*Mailotrichocera*	*Mailotrichocera jurassica*	Kalugina	1985	Uppermost middle or earliest Upper Jurassic	Uda, E. Transbaikalia
	*Mailotrichocera gracilis*	Krzemińska, Krzemiński & Dahl	2009a	Jurassic/Cretaceous boundary	Daya, Unda and Shevia, E Transbaikalia
	*Mailotrichocera mikereichi*	Krzemińska, Krzemiński & Ansorge	2009a	Lower Jurassic	Dobbertin, Germany; Grimmen, Germany
	*Mailotrichocera ovifera*	Krzemińska, Krzemiński & Dahl	2009a	Jurassic/ Cretaceous boundary	Unda, E. Transbaikalia
	*Mailotrichocera prisca*	Krzemińska, Krzemiński & Dahl	2009a	Jurassic/Cretaceous boundary	Unda and Shevia, E. Transbaikalia
	*Mailotrichocera sukachevae*	Krzemińska, Krzemiński & Dahl	2009a	Jurassic/Cretaceous boundary	Unda, E Transbaikalia
	*Mailotrichocera variabilis*	Krzemińska, Krzemiński & Ansorge	2009a	Lower Jurassic	Dobbertin, Germany; Grimmen, Germany
	*Mailotrichocera zessini*	Krzemińska, Krzemiński & Ansorge	2009a	Lower Jurassic	Grimmen, Germany
*Paleotrichocera*	*Paleotrichocera mongolica*	Kalugina	1986	Lower Cretaceous	Gurvan Erenyi Nuru, Mongolia
*Paracladura*	*Paracladura caucasiana*	Krzemińska, Krzemiński & Dahl	2009a	Middle Miocene	Stavropol, Caucasus
*Rasnitsynina*	*Rasnitsynina collecta*	Krzemińska, Krzemiński & Dahl	2009a	Jurassic/Cretaceous boundary	Shevia, E. Transbaikalia
	*Rasnitsynina minae*	Krzemińska, Krzemiński & Dahl	2009a	Jurassic/Cretaceous boundary	Shevia and Daya, E Transbaikalia
*Tanyochoreta (Sinotrichocera)*	*Tanyochoreta (Sinotrichocera) parva*	Zhang	2006	Middle Jurassic	Daohugou, China
*Tanyochoreta (Tanyochoreta)*	*Tanyochoreta (Tanyochoreta) chifengica*	Zhang	2006	Middle Jurassic	Daohugou, China
	*Tanyochoreta (Tanyochoreta) integera*	Zhang	2006	Middle Jurassic	Daohugou, China
*Tanyochoreta (Trichokara)*	*Tanyochoreta (Trichokara) composita*	Krzemińska, Krzemiński & Dahl	2009a	Upper Jurassic	Karatau, Kazakhstan
	*Tanyochoreta (Trichokara) fracta*	Krzemińska, Krzemiński & Dahl	2009a	Upper Jurassic	Karatau, Kazakhstan
	*Tanyochoreta (Trichokara) minuta*	Krzemińska, Krzemiński & Dahl	2009a	Upper Jurassic	Karatau, Kazakhstan
	*Tanyochoreta (Trichokara) tenuis*	Krzemińska, Krzemiński & Dahl	2009a	Upper Jurassic	Karatau, Kazakhstan
	*Tanyochoreta (Trichokara) zagadka*	Krzemińska, Krzemiński & Dahl	2009a	Jurassic/Cretaceous boundary	Unda, E Transbaikalia
	*Tanyochoreta (Trichokara) zbulwami*	Krzemińska, Krzemiński & Dahl	2009a	Jurassic/Cretaceous boundary	Daya and Unda, E Transbaikalia
*Trichocera*	*Trichocera scudderi*	Meunier	1915	Upper Oligocene	Rott, Germany
	*Trichocera miocaenica*	Statz	1934	Upper Oligocene	Rott, Germany
	*Trichocera antiqua*	Dahl	1971	Upper Eocene	Baltic
	*Trichocera primaeva*	Dahl	1971	Upper Eocene	Baltic
	*Trichocera fujiyamai*	Gentilini	1984	Upper Miocene	Monte Castellaro, Italy
	*Trichocera anbar*	Podenas	2001	Upper Eocene	Baltic
	*Trichocera bona*	Podenas	2001	Upper Eocene	Baltic
	*Trichocera cerea*	Podenas	2001	Upper Eocene	Baltic
	*Trichocera diluta*	Podenas	2001	Upper Eocene	Baltic
	*Trichocera ebenos*	Podenas	2001	Upper Eocene	Baltic
	*Trichocera christelae*	Krzemińska, Krzemiński & Dahl	2009a	Upper Eocene	Baltic
	*Trichocera corami*	Krzemińska, Krzemiński & Dahl	2009a	Lower Cretaceous	Purbeck, UK
	*Trichocera cretacea*	Krzemińska, Krzemiński & Dahl	2009a	Lower Cretaceous	Baissa, Russia
	*Trichocera hanswerneri*	Krzemińska, Krzemiński & Dahl	2009a	Upper Eocene	Baltic
	*Trichocera turgana*	Krzemińska, Krzemiński & Dahl	2009a	earlier Lower Cretaceous	Turga, E. Transbaikalia
*Undaya*	*Undaya alata*	Krzemińska, Krzemiński & Dahl	2009a	Jurassic/Cretaceous boundary	Unda and Shevia, E. Transbaikalia
	*Undaya comis*	Krzemińska, Krzemiński & Dahl	2009a	Jurassic/Cretaceous boundary	Unda, E. Transbaikalia
	*Undaya gargantuina*	Krzemińska, Krzemiński & Dahl	2009a	Jurassic/Cretaceous boundary	Daya and Unda, E. Transbaikalia
	*Undaya hilara*	Krzemińska, Krzemiński & Dahl	2009a	Jurassic/Cretaceous boundary	Unda and Shevia, E. Transbaikalia
	*Undaya kaluginae*	Krzemińska, Krzemiński & Dahl	2009a	Jurassic/Cretaceous boundary	Daya, E. Transbaikalia
	*Undaya lenae*	Krzemińska, Krzemiński & Dahl	2009a	Upper Jurassic	Shar-Teg, Mongolia
	*Undaya lukashevichae*	Krzemińska, Krzemiński & Dahl	2009a	Upper Jurassic	Shar-Teg, Mongolia
	*Undaya maxima*	Krzemińska, Krzemiński & Dahl	2009a	Lower Cretaceous	Kempendyay, Russia
	*Undaya mitis*	Krzemińska, Krzemiński & Dahl	2009a	Jurassic/Cretaceous boundary	Daya and Unda, E. Transbaikalia
	*Undaya molesta*	Krzemińska, Krzemiński & Dahl	2009a	Jurassic/Cretaceous boundary	Unda and Daya, E. Transbaikalia
	*Undaya namdyriensis*	Krzemińska, Krzemiński & Dahl	2009a	Lower Cretaceous	Kempendyay, Russia
	*Undaya parvula*	Krzemińska, Krzemiński & Dahl	2009a	Jurassic/Cretaceous boundary	Daya and Unda, E. Transbaikalia
	*Undaya pura*	Krzemińska, Krzemiński & Dahl	2009a	Jurassic/Cretaceous boundary	Unda and Daya, E. Transbaikalia
	*Undaya salsa*	Krzemińska, Krzemiński & Dahl	2009a	Lower Cretaceous	Kempendyay, Russia
	*Undaya savina*	Krzemińska, Krzemiński & Dahl	2009a	Jurassic/Cretaceous boundary	Savina, E. Transbaikalia
	*Undaya saxea*	Krzemińska, Krzemiński & Dahl	2009a	Lower Cretaceous	Kempendyay, Russia
	*Undaya triangula*	Krzemińska, Krzemiński & Dahl	2009a	Lower Cretaceous	Kempendyay, Russia
*Zherikhinina*	*Zherikhinina itatica*	Kalugina	1985	Middle Jurassic	Kubekovo, Russia
	*Zherikhinina alastos*	Krzemińska & Lukashevich	2009b	Upper Jurassic	Shar Teg, Mongolia
	*Zherikhinina baissana*	Krzemińska, Krzemiński & Dahl	2009a	Lower Cretaceous	Baissa, Russia
	*Zherikhinina bontsagana*	Krzemińska, Krzemiński & Dahl	2009a	Lower Cretaceous	Bon Tsagan, Mongolia
	*Zherikhinina karatavica*	Krzemińska, Krzemiński & Dahl	2009a	Upper Jurassic	Karatau, Kazakhstan
	*Zherikhinina novospasskaya*	Krzemińska, Krzemiński & Dahl	2009a	later Lower or early Middle Jurassic	Novospasskoe, Russia
	*Zherikhinina reni*	Krzemińska, Krzemiński & Dahl	2009a	Middle Jurassic	Daohugou, China
	*Zherikhinina tola*	Krzemińska, Krzemiński & Dahl	2009a	Lower Cretaceous	Onokhoy, Mongolia
	*Zherikhinina zherikhini*	Krzemińska, Krzemiński & Dahl	2009a	Upper Jurassic	Karatau, Kazakhstan

The specimens for this study were collected from the Jiulongshan Formation of the Daohugou Village in Inner Mongolia, China. The Daohugou fossil-bearing beds are considered as the late Middle Jurassic (Bathonian-Callovian boundary, 165 Mya) (Ren et al. 2002; Gao and Ren 2006; Ren et al. 2010a; Shi et al. 2011). Daohugou is one of the localities where the fossils of Yanliao biota were distributed. A huge number of fossil insects have been reported (Ren and Engel 2007; Engel and Ren 2008; Liu and Ren 2008; Ren et al. 2009; Wang and Ren 2009; Gu et al. 2010; Ren et al. 2010b; Wang et al. 2010; Wang et al. 2012; Yang et al. 2012).

## Materials and methods

The wing venation nomenclature used in this paper is based on the interpretations and system proposed by Lukashevich (2004) and Krzemińska et al. (2009a). The fossil specimens were examined under a Leica MZ7.5 dissecting microscope and illustrated with the aid of a drawing tube attachment. Line drawings were prepared with Adobe Photoshop CS3 Extended graphics software.

All specimens studied in the paper are housed in the Key Lab of Insect Evolution and Environmental Changes, College of Life Sciences, Capital Normal University, Beijing, China.

## Systematic paleontology

### Family Trichoceridae Kertész, 1902
Genus *Eotrichocera* Kalugina, 1985

#### Subgenus Archaeotrichocera

Krzemińska, Krzemiński & Dahl, 2009

##### Type species.

*Eotrichocera (Archaeotrichocera) ephemera* Zhang, 2006

##### Other included species.

*Eotrichocera (Archaeotrichocera) conica* Krzemińska, Krzemiński & Ren, 2009; *Eotrichocera (Archaeotrichocera) rara* Krzemińska, Krzemiński & Ren, 2009; *Eotrichocera (Archaeotrichocera) spatiosa* Liu, Shih & Ren, 2012.

##### Key to the species of *Eotrichocera (Archaeotrichocera)*

**Table:** 

1	Sc ending at anterior margin distad of R_2_	2
–	Sc ending at anterior margin proximad of R_2_	3
2	Large size (wing length 12.0 mm)	*Eotrichocera (Archaeotrichocera) spatiosa* Liu, shih & Ren, 2012 (Daohugou, J_2_)
–	Medium size (wing length 5.5 mm)	*Eotrichocera (Archaeotrichocera) rara* Krzemińska, Krzemiński & Ren, 2009 (Daohugou, J_2_)
3	Crossvein sc-r distad of 1/2 (at 2/3) of length of Rs	4
–	Crossvein sc-r proximad of or at 1/3 of length of Rs	5
4	Rs forking distad of 2/3 (at 0.77) times of wing length	*Eotrichocera (Archaeotrichocera) ephemera* Zhang, 2006 (Daohugou, J_2_)
–	Rs forking proximad of 2/3 (at about 0.53) times of wing length	*Eotrichocera (Archaeotrichocera) longensis* sp. n. (Daohugou, J_2_)
5	A_2_ long (0.22 times as long as wing), d cell narrow and long (W/L=0.43	*Eotrichocera (Archaeotrichocera) conica* Krzemińska, Krzemiński & Ren, 2009 (Daohugou, J_2_)
–	A_2_ short (0.13 times as long as wing), d cell broad (W/L=0.58)	*Eotrichocera (Archaeotrichocera) amabilis* sp. n. (Daohugou, J_2_)

#### Eotrichocera (Archaeotrichocera) longensis sp. n.

http://zoobank.org/8A0D358E-7BCA-476B-A0A9-6EECA93FCBA8

http://species-id.net/wiki/Eotrichocera_longensis

##### Etymology.

“*longensis*” is a Latin word, referring to the long leg of this specimen.

##### Diagnosis.

Sc rather short about 0.65 times as long as the wing and ending at anterior margin proximad of R_2_; Rs forking proximad of 2/3 (at about 0.55) times of wing length; the d-cell narrow and long (about 2.5 times as long as wide); A_2_ short and bending sharply toward anterior margin (angle about 128°).

##### Holotype.

**Figure 1.**
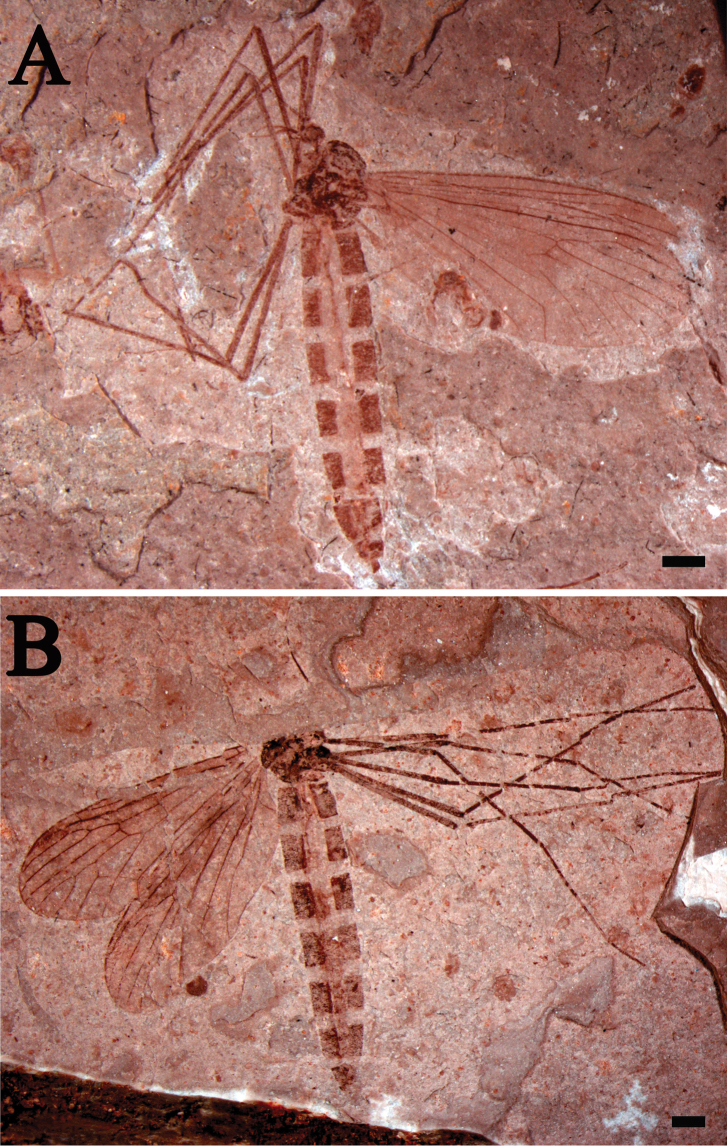
*Eotrichocera (Archaeotrichocera) longensis* sp. n. Holotype, specimen CNU-DIP-NN2013133 **A** Photograph. Paratype, specimen CNU-DIP-NN2013131 **B** Photograph. Scale bars = 1 mm.

**Figure 2.**
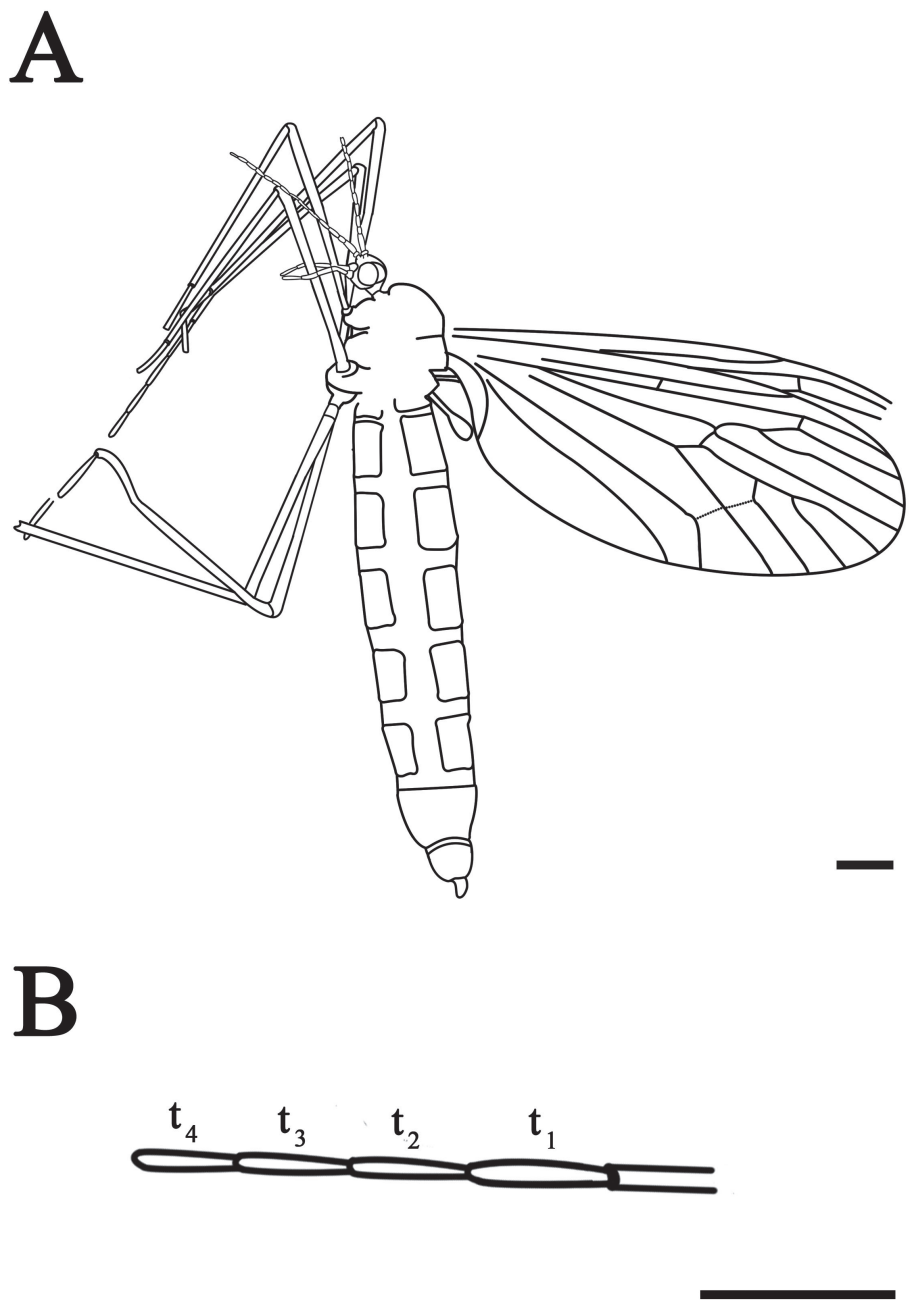
*Eotrichocera (Archaeotrichocera) longensis* sp. n. Holotype, specimen CNU-DIP-NN2013133 **A** Line drawing **B** Tarsus of the mid leg. Scale bars = 1 mm; t_1_ = the first segment of tarsus; t_2_ = the second segment of tarsus; t_3_ = the third segment of tarsus; t_4_ = the fourth segment of tarsus.

**Figure 3.**
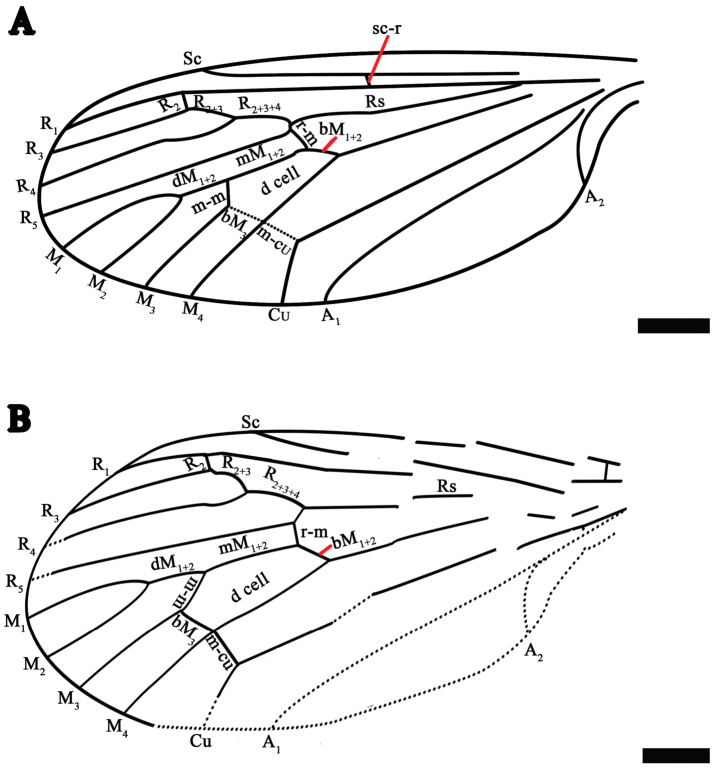
*Eotrichocera (Archaeotrichocera) longensis* sp. n. Holotype, specimen CNU-DIP-NN2013133 **A** Line drawing of left wing. Paratype, specimen CNU-DIP-NN2013131 **B** Line drawing of left wing. Scale bars = 1 mm

An almost complete female specimen with well-preserved body, wings and head. Specimen number CNU-DIP-NN2013133. Wing length 9.0 mm, width 3.8 mm (Figs 1A, 2, 3A).

##### Paratype.

A female specimen with body and wings, specimen number CNU-DIP-NN2013131. Wing length 7.7 mm, width 3 mm (Figs 1B, 3B).

##### Locality and horizon.

Jiulongshan Formation, Late Middle Jurassic, Daohugou Village, Ningcheng County, Inner Mongolia Autonomous Region, China.

##### Description.

Based on Holotype, different characters of the paratype CNU-DIP-NN2013131 in brackets. Medium-sized winter crane flies, body length (including head) 13 mm with well preserved wings, body and head, [paratype body length (excluding head) 10.5 mm].

*Head*: Antenna very long, about 3.5 times as long as the head length, palpi about two times as long as the head length, compound eyes preserved.

*Thorax*: Much higher, in lateral view, than that of the abdomen, subcircular in shape, with robust and well-developed mesonotum. The halters spoon-type and the length of halters as long as thorax.

*Wings*: Wing is shorter than abdomen, not covering the end of the abdomen. Length 9.0 mm [Paratype with wing length of 7.7 mm], narrow and long (about 2.5 times as long as wide); venation clear, Sc rather short about 0.68 times as long as the wing [Paratype Sc rather short, about 0.65 times as long as the wing] and ending at anterior margin proximad of R_2_; crossvein sc-r locating at 2/3 of Rs; Rs arising about one-fifth from the base of the wing; R_2+3_ about 0.8 times as long as R_2+3+4_; R_2_ about one-tenth of length of R_3_; R_3_ almost three times as long as the R_2+3_; dM_1+2_ 0.6 times as long as mM_1+2_, while M_1_ 2.5 times of the dM_1+2_; a well developed m-m crossvein about three-fourth length of bM_3_, closing the d-cell and nearly 0.3 length of d-cell; bM_1+2_ nearly 1.0 times as long as the length of the r-m and the latter at one-fifth of the d-cell; d-cell narrow and long (about 2.5 times as long as wide) and almost one-fifth length of wing; both crossveins m-m and m-cu intersecting with M_4_ at the same point; Cu long, curved (angle about 135°) and reaching the wing posterior margin at 0.6 from the base of the wing; the stem of A divided into A_1_ and A_2_; A_1_ long, slightly curving and reaching the wing posterior margin; A_2_ short, 0.15 (right wing) [Paratype 0.14] times as long as wing and almost 0.3 times as long as length of A_1_, bending sharply (angle about 128°) and reaching the wing posterior margin.

*Legs*: Legs slender and long; the hind leg nearly 1.2 times as long as the abdomen and 1.3 times as long as the wing. Tarsus with five segments; the first segment of tarsus (t_1_) is 1.2 times as long as t_2_ in mid leg.

*Abdomen*: Abdomen relatively long and thin, with ten segments. Female genital discernible.

##### Remarks.

*Eotrichocera (Archaeotrichocera) longensis* sp. n. is assigned to Thrichocerinae based on the following characters: d-cell medium, m-cu present; A_2_ short, antennae long, flagellomeres thin, much longer than two times of the head length. It belongs to *Eotrichocera (Archaeotrichocera)* because of wing length from 7.7 to 9.0 mm and d-cell almost one-fifth of wing length. In addition, it differs from all other known Thrichocerinae by its A_2_ rather short and bending sharply toward anterior margin (angle about 128°), R_2_ relatively long, Sc forking proximad of 2/3 (at about 0.55) times of wing length, and d-cell narrow and long. To compare the key characters among the new species and other species of *Eotrichocera (Archaeotrichocera)*, we set up the Table 2.

**Table 2.** Comparison of key characters among the two new species and other species of *Eotrichocera (Archaeotrichocera)* Krzemińska, Krzemiński & Dahl, 2009. L/W = ratio of length/width; W/L = ratio of width/length.

Key characters	*Eotrichocera (Archaeotrichocera) ephemera* Zhang, 2006	*Eotrichocera (Archaeotrichocera) conica* Krzemińska, Krzemiński & Ren, 2009	*Eotrichocera (Archaeotrichocera) rara* Krzemińska, Krzemiński & Ren, 2009	*Eotrichocera (Archaeotrichocera) spatiosa* Liu, Shih & Ren, 2012	*Eotrichocera (Archaeotrichocera) longensis* sp. n.	*Eotrichocera (Archaeotrichocera) amabilis* sp. n.
Wing length, in mm and (L/W)	7.1 (L/W=2.8)	10.0 (L/W=3)	5.5	12.0 (L/W=2.7)	9.0 (L/W=2.3)	5.2 (L/W=2.2)
Sc length and ending at anterior margin	0.77 times of wing length, ending proximad of R_2_	0.71 times of wing length, ending proximad of R_2_	0.77 times of wing length, ending distad of R_2_	0.84 times of wing length, and ending distad of R_2_	about 0.65 times of wing length, ending proximad of R_2_	about 0.71 times of wing length, ending proximad of R_2_
sc-r position	at 2/3 of length of Rs	at 1/3 of length of Rs	at 1/2 of length of Rs	at 2/3 of length of Rs	at 2/3 of length of Rs	at 1/3 of length of Rs
Position of Rs forking	0.77 times of wing length	0.64 times of wing length	0.57 times of wing length	0.58 times of wing length	0.53 times of wing length	0.55 times of wing length
d-cell W/L (length)	W/L=0.53 (1/6 of wing length)	W/L=0.43 (0.2 times of wing length)	W/L=0.39 (0.21 times of wing length)	W/L=0.47 (0.19 times of wing length)	W/L= 0.4 (almost 1/5 of wing length)	W/L=0.58 (almost 0.17 of wing length)
A_2_ length	long (about 1/4 of wing length), curved evenly to posterior margin	medium (0.22 times of wing length), not reaching posterior margin	rather short (1/5 of wing length) and not reaching posterior margin	short (about 0.21 times of wing length) and curving to posterior margin	short (0.14) times of wing length) and curved to posterior margin	short (0.13) times of wing length) and curved to posterior margin
r-m length	.......	1/5 of length of d-cell	.......	about 1/3 of length of d-cell	1/5 of length of the d-cell	0.24 or 0.15 of length of the d-cell

#### Eotrichocera (Archaeotrichocera) amabilis sp. n.

http://zoobank.org/D32A4E4B-EDF1-4684-802E-92F9617DEAB2

http://species-id.net/wiki/Eotrichocera_amabilis

##### Etymology.

The specific name of “*amabilis*” is a Latin word, meaning lovely.

##### Diagnosis.

Body small and wing short; Sc 0.71 times as long as wing; the d-cell broad (about 1.7 times as long as wide); A_2_ short and bending sharply toward posterior margin (angle about 128°).

##### Holotype.

**Figure 4.**
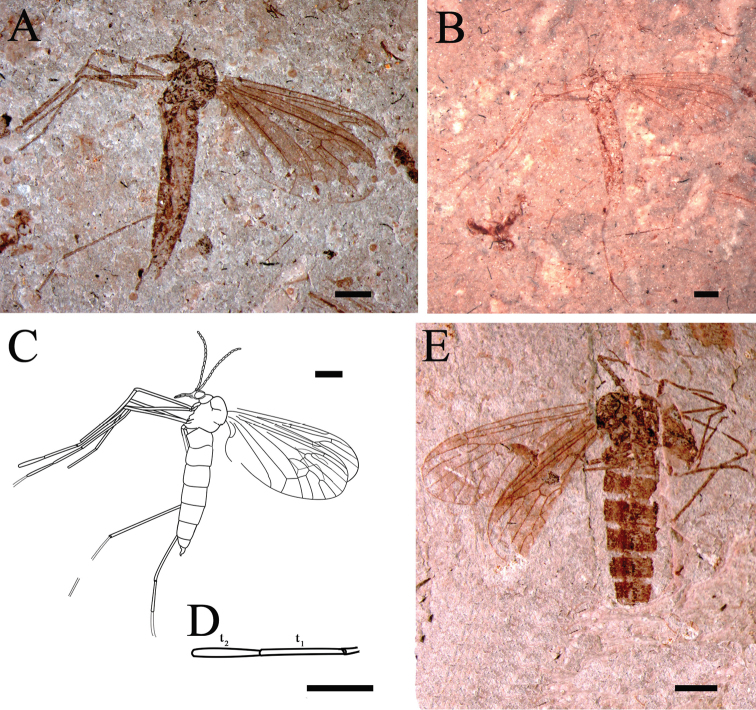
*Eotrichocera (Archaeotrichocera) amabilis* sp. n. Holotype, specimen CNU-DIP-NN2013134 **A** Photograph **B** Photograph, under alcohol **C** Line drawing **D** Tarsus of the mid leg. Paratype, specimen CNU-DIP-NN2013132 **E** Photograph. Scale bars = 1 mm; t_1_ = the first segment of tarsus; t_2_ = the second segment of tarsus.

**Figure 5.**
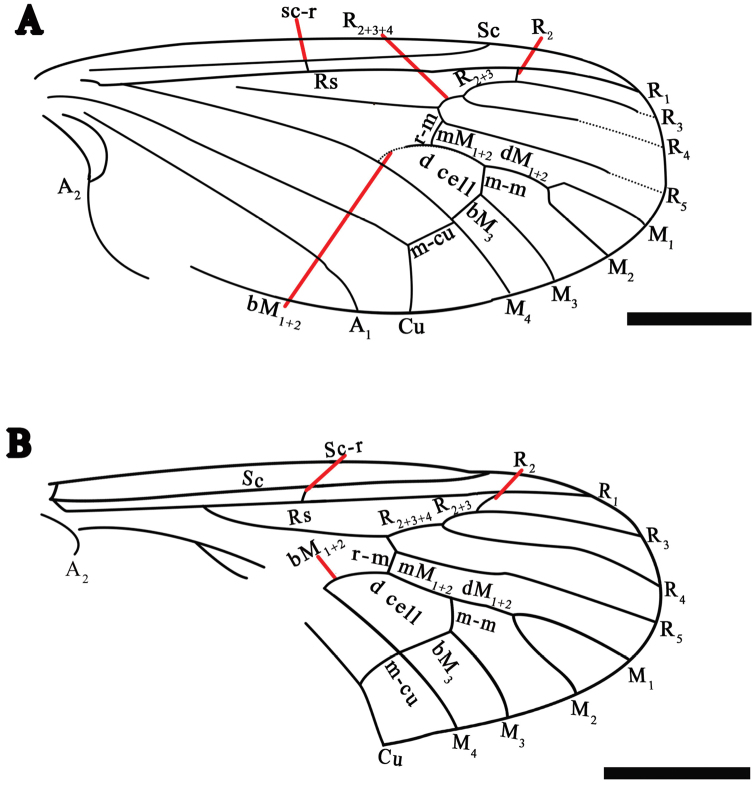
*Eotrichocera (Archaeotrichocera) amabilis* sp. n. Holotype, specimen CNU-DIP-NN2013134 **A** Line drawing of left wing. Paratype, specimen CNU-DIP-NN2013132 **B** Line drawing of left wing. Scale bars = 1 mm.

**Figure 6.**
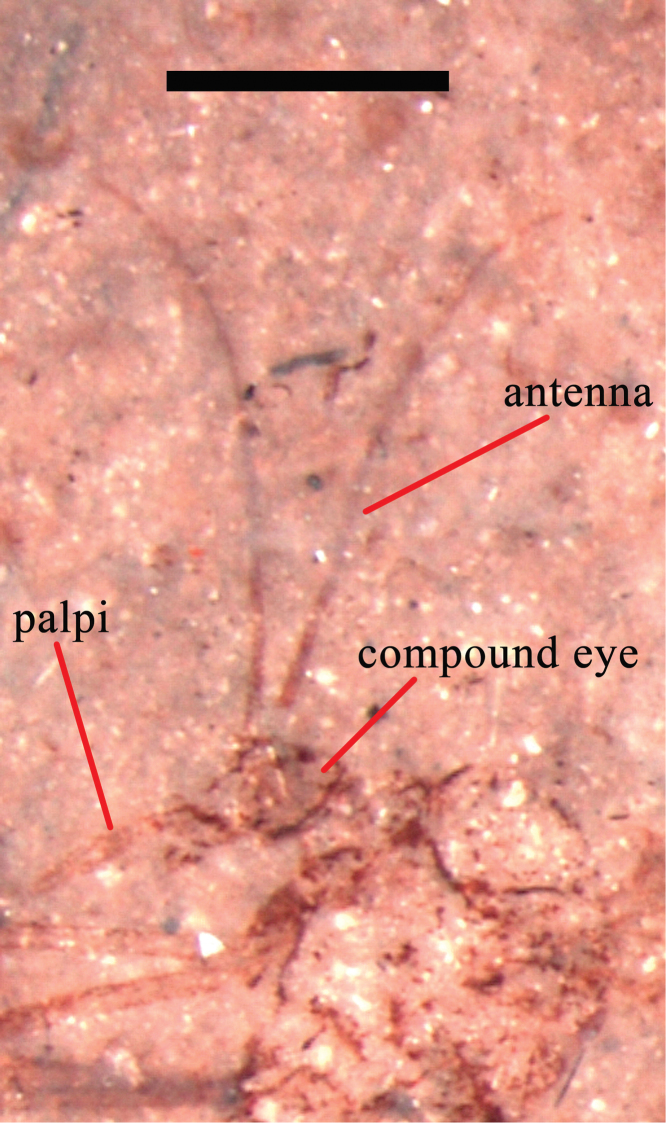
*Eotrichocera (Archaeotrichocera) amabilis* sp. n. Holotype, specimen CNU-DIP-NN2013134, Photograph of head, under alcohol. Scale bar = 1 mm.

An almost complete female specimen with well-preserved body, wings and head. Specimen number CNU-DIP-NN2013134, Wing length 5.2 mm, width 2.2 mm (Figs 4A–D, 5A, 6).

##### Paratype.

A specimen with body and wings with partial venation, specimen number CNU-DIP-NN2013132, (Figs 4E, 5B).

##### Locality and horizon.

Jiulongshan Formation, Late Middle Jurassic, Daohugou Village, Ningcheng County, Inner Mongolia Autonomous Region, China.

##### Description.

Based on Holotype, different characters of the paratype CNU-DIP-NN2013132 in brackets. Medium-sized winter crane flies, head length 0.47 mm, body length (including head) 5.8 mm with well preserved body and wings. [Paratype with partial body and wings with partial venation].

*Head*: antenna very long, about 5.7 times as long as the head length, palpi about two times as long as the head length, compound eyes preserved (Figs 4C, 6).

*Thorax*: Much higher, in lateral view, than that of the abdomen, subcircular in shape, with robust and well-developed mesonotum.

*Wings*: Wing is shorter than abdomen, not covering the end of the abdomen. Wing length of 5.2 mm [Paratype with wing length 5.0 mm], narrow and long (L/W=2.2); venation clear, Sc rather short about 0.71 times as long as the wing and terminating clearly proximad of R_2_; crossvein sc-r locating at 1/3 [Paratype 1/2] of Rs, and distad to the Sc ending; [Paratype Rs arising about one-fourth from the base of the wing]; Rs forking at 0.55 [Paratype 0.64] times of wing length; R_2+3_ about 1.9 times as long as R_2+3+4_; R_2_ about 0.18 of length of R_3_; R_3_ almost 3.7 times as long as the R_2+3_; R_5_ 9.0 times as long as R_2+3+4_; M_1_ 1.6 times of the dM_1+2_; crossvein m-m well developed about 0.73 times as long as bM_3_, closing the d-cell and nearly 0.17 [Paratype 0.21] times as long as wing; bM_1+2_ nearly 2.1 times as long as the length of the r-m and the latter at one-fourth of the d-cell; d-cell broad (W/L=0.58 [Paratype 0.56]) and almost 0.17 times of length of wing; Cu long, curved (angle about 121°) and reaching the wing posterior margin at 0.67 from the base of the wing; the stem of A divided into A_1_ and A_2_; A_1_ long, slightly curving and reaching the wing posterior margin; A_2_ short, 0.13 times as long as wing and almost 0.25 times as long as length of A_1_, bending sharply and reaching the wing posterior margin.

*Abdomen*: Abdomen relatively long and thin, with ten segments. Female genitalia discernible (Figs 4A–C) [Paratype genitalia indiscernible].

*Legs*: Legs slender and long; the hind leg nearly 1.5 times as long as the abdomen and 1.4 times as long as the wing. Tarsus with five segments; the first segment of tarsus (t_1_) is 1.2 times as long as t_2_ in mid leg.

##### Remarks.

The new species is compared and differentiated from all other species in *Eotrichocera (Archaeotrichocera)* in Table 2.

Due to limitation of fossil preservation, some of the morphological characters of previously described fossil are not objective or clear. We set up an aforementioned key based on the Sc length and ending location at anterior margin, wing length, crossvein sc-r position, Rs forking location and A_2_ length, to differentiate the species of subgenus *Archaeotrichocera*. These characters may help future morphological and taxonomic studies in differentiating fossil species of Trichoceridae.

## Supplementary Material

XML Treatment for Archaeotrichocera

XML Treatment for Eotrichocera (Archaeotrichocera) longensis

XML Treatment for Eotrichocera (Archaeotrichocera) amabilis

## References

[B1] Dahl C Alexander C-P (1976) A world catalogue of Trichoceridae Kertész, 1902 (Diptera). Entomol Scand 7: 7-18. doi: 10.1163/187631276X00027

[B2] Engel MS Ren D (2008) New snakeflies from the Jiulongshan Formation of Inner Mongolia, China (Raphidioptera). Journal of the Kansas Entomological Society 81(3): 188-193. doi: 10.2317/JKES-802.19.1

[B3] Gao K-Q Ren D (2006) Radiometric Dating of Ignimbrite from Inner Mongolia Provides no Indication of a Post-Middle Jurassic Age for the Daohugou Beds. Acta Geologica Sinica (English Edition) 80: 42-45. doi: 10.1111/j.1755-6724.2006.tb00793.x

[B4] Gu J-J Qiao G-X Ren D (2010) Revision and new taxa of fossil Prophalangopsidae (Orthoptera: Ensifera). Journal of Orthoptera Research 19(1): 41-56. doi: 10.1665/034.019.0110

[B5] Hågvar S Krzemińska E (2007) Contribution to the winter phenology of Trichoceridae (Diptera) in snow-covered southern Norway. Stud Dipt 14: 271-283

[B6] Kalugina NS Kovalev VG (1985) Diptera Insects of Jurassic of Siberia. Nauka, Moscow, 198 pp. [In Russian]

[B7] Krzemińska E Krzemiński W Dahl C (2009a) Monograph of Fossil Trichoceridae (Diptera) over 180 Million Years of Evolution. Institute of Systematics and Evolution of Animals of the Polish Academy of Sciences Press, Krakow, Poland, 35–40

[B8] Krzemińska E Lukashevich E (2009b) The Late Jurassic fossil from Asia ancestory to a recent genus, Nothotrichocera Alexander 1926 (Diptera: Trichoceridae). Zoosymposia 3: 103-110

[B9] Liu L-X Shih C-K Ren D (2012) Two new species of Ptychopteridae and Trichoceridae from the Middle Jurassic of northeastern China (Insecta: Diptera: Nematocera). Zootaxa 3501: 55-62

[B10] Liu Y-S Ren D (2008) Two new Jurassic stoneflies (Insecta: Plecoptera) from Daohugou, Inner Mongolia, China. Progress in Natural Science 18: 1039-1042. doi: 10.1016/j.pnsc.2008.03.014

[B11] Lukashevich ED (2004) A revision of the Genus *Eoptychopterina* (Diptera: Eoptychopteridae). Paleontological Journal 38(3): 294-306

[B12] Ren D Gao K-Q Guo Z-G Ji S-A Tan J-J Song Z (2002) Stratigraphic division of the Jurassic in the Daohugou Area, Ningcheng, Inner Mongolia. Geological Bulletin of China 21: 584-591

[B13] Ren D Engel MS (2007) A split-footed lacewing and two episomylines from the Jurassic of China (Neuroptera). Annales Zoologici (Warszawa) 57(2): 211-219

[B14] Ren D Labandeira CC Santiago-Blay JA Rasnitsyn AP Shih C-K Bashkuev A Logan MAV Hotton CL Dilcher D (2009) A probable pollination mode before angiosperms: Eurasian, long-proboscid scorpionflies. Science 326: 840-847. doi: 10.1126/science.1178338 19892981 10.1126/science.1178338 PMC2944650

[B15] Ren D Shih C-K Gao T-P Yao Y-Z Zhao Y-Y (2010a) Silent Stories – Insect Fossil Treasures from Dinosaur Era of the Northeastern China. Science Press, Beijing, 324 pp.

[B16] Ren D Shih C-K Labandeira CC (2010b) New Jurassic Pseudopolycentropodids from China (Insecta: Mecoptera). Acta Geologica Sinica (English Edition) 84(1): 22-30. doi: 10.1111/j.1755-6724.2010.00166.x

[B17] Shi C-F Yang Q Ren D (2011) Two new fossil lacewing species from the Middle Jurassic of Inner Mongolia, China (Neuroptera: Grammolingiidae). Acta Geologica Sinica (English Edition) 85(2): 482-489. doi: 10.1111/j.1755-6724.2011.00416.x

[B18] Wang Y-J Liu Z-Q Wang X Shih C-K Zhao Y-Y Engel MS Ren D (2010) Ancient pinnate leaf mimesis among lacewings. Proceedings of the National Academy of Sciences USA 107(37): 16212-16215. doi: 10.1073/pnas.1006460107 10.1073/pnas.1006460107 PMC2941307 20805491

[B19] Wang Y-J Labandeira CC Shih C-K Ren D Ding Q-L Wang C Zhao Y-Y (2012) Jurassic mimicry between a hanging fly and a ginkgo from China. Proceedings of The National Academy of Sciences USA 109(50): 20514-10519. doi: 10.1073/pnas.1205517109 10.1073/pnas.1205517109 PMC3528590 23184994

[B20] Wang Y Ren D (2009) New fossil palaeontinids from the Middle Jurassic of Daohugou, Inner Mongolia, China (Insecta, Hemiptera). Acta Geologica Sinica (English Edition) 83(1): 33-38. doi: 10.1111/j.1755-6724.2009.00004.x

[B21] Yang D (2009) Diptera. In: Yang D (Ed) Fauna of Hebei, China.Agricultural Science and Technology Press, Beijing, China: 14-25

[B22] Yang Q Makarkin VN Winterton SL Khramov AV Ren D (2012) A remarkable new family of Jurassic insects (Neuroptera) with primitive wing venation and its phylogenetic position in neuropterida. PLoS ONE 7(9): . doi: 10.1371/journal.pone.0044762 10.1371/journal.pone.0044762 PMC3445537 23028608

[B23] Yang J-K Yang D (1995) Insecta: Diptera: Trichoceridae. In: Yang J-K Yang D (Eds) Insects and Large-Scale Fungi in Gutianshan of Zhejiang Province. Zhejiang Science and Technology Press, Zhejiang, China, 175-179

[B24] Zhang J-F (2006) New winter crane flies (Insecta: Diptera: Trichoceridae) from the Jurassic Daohugou Formation (Inner Mongolia, China) and their associated biota. Canadian Journal of Earth Sciences 43: 9-22. doi: 10.1139/e05-092

